# Adaptive Scheme of Denoising Autoencoder for Estimating Indoor Localization Based on RSSI Analytics in BLE Environment

**DOI:** 10.3390/s23125544

**Published:** 2023-06-13

**Authors:** Kyuri Kim, Jaeho Lee

**Affiliations:** 1 Department of IT Media Engineering, Duksung Women’s University, Seoul 01369, Republic of Korea; 00a3d2@duksung.ac.kr; 2 Department of Software, Duksung Women’s University, Seoul 01369, Republic of Korea

**Keywords:** RSSI, indoor localization, neural networks, denoising autoencoder

## Abstract

In indoor environments, estimating localization using a received signal strength indicator (RSSI) is difficult because of the noise from signals reflected and refracted by walls and obstacles. In this study, we used a denoising autoencoder (DAE) to remove noise in the RSSI of Bluetooth Low Energy (BLE) signals to improve localization performance. In addition, it is known that the signal of an RSSI can be exponentially aggravated when the noise is increased proportionally to the square of the distance increment. Based on the problem, to effectively remove the noise by adapting this characteristic, we proposed adaptive noise generation schemes to train the DAE model to reflect the characteristics in which the signal-to-noise ratio (SNR) considerably increases as the distance between the terminal and beacon increases. We compared the model’s performance with that of Gaussian noise and other localization algorithms. The results showed an accuracy of 72.6%, a 10.2% improvement over the model with Gaussian noise. Furthermore, our model outperformed the Kalman filter in terms of denoising.

## 1. Introduction

Location-based services (LBS) are essential technologies in various industries. The Global Positioning System (GPS) is typically used for outdoor localization. However, GPS cannot be employed indoors because microwaves cannot be received indoors owing to disturbances derived from roofs, walls, and other objects. Therefore, other wireless signals, such as Wi-Fi and Bluetooth, have been used for indoor positioning [1,2,3,4]. Angle- and time-based methods for indoor localization systems use wireless signals. Angle-of-arrival (AoA) [5] systems are based on the angle difference between the signals received from two or more antennas. The time-of-arrival (ToA) [6] technique is a time-based algorithm that uses the arrival time of a signal from a user device to several beacons. Other studies have approached localization using radar or visible light [7,8]. However, these methods are affected by the multipath effect of signals caused by reflections and interference in indoor environments. Therefore, fingerprinting [9] based on a received signal strength indicator (RSSI) has been widely used for indoor localization.

**Figure 1:**
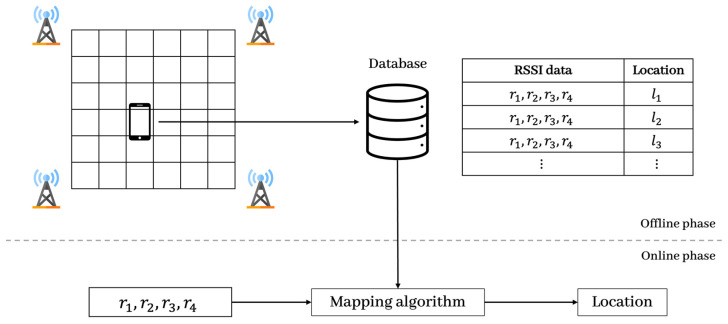
Fingerprinting-based localization system.

Fingerprinting is based on data collected from every location in an area. Learning-based fingerprinting has two phases: offline (training) and online (positioning), as shown in Figure 1. The offline phase is the process of constructing a fingerprint database (radio map) by collecting the RSSI received from several access points (AP) to reference points (RP). In the online phase, the location of an unknown point is estimated as the cell with the most similar data by comparing the RSSI measured by the user device with the fingerprints in the database. Thus, a mapping algorithm that converts an RSSI into location information can utilize various mapping-based algorithms, such as the K-nearest neighbor (KNN) and support vector machine (SVM), or deep neural networks.

In general, an RSSI cannot be satisfactory for estimating an indoor position from a fingerprinting perspective. The values of RSSIs measured on a site present high variations due to noise, regardless of communication interfaces, especially in indoor environments. Such noise is typically made from stationary obstacles, e.g., walls, roofs, floors, and furniture, as well as fixed objects, and so there can be a feature, depending on the position. Thus, learning-based approaches have been used to find a feature for a specific indoor position. Referring to [10,11], feature-based approaches provide some computationally expensive points, and they can create problems in cloudless systems, e.g., connection-poor areas, and in-time order environments. The positional relation tracking on an object’s movement must be also handled, and it must be considerable to employ learning methods with low computational costs and in-time requirements. From this viewpoint, an encoder of auto encoder without CNN may be a reasonable solution.

However, in an indoor environment, RSSI data are sensitive to noise owing to signal interference caused by walls and obstacles. The signal-to-noise ratio (SNR) increases as the distance between the AP and the user device increases. A noise-resistant model is required to achieve high indoor positioning accuracy through removing noise. In addition, it is necessary to consider that the SNR will vary with distance. Hence, above the value of an RSSI, the amount of RSSI variations according to the corresponding distances must be considered to obtain a feature. In this paper, we propose a new scheme based on a variable denoising autoencoder (DAE) [12] to enhance the accuracy of indoor localization systems with adaptive noise generation schemes.

The learning process of the DAE includes the addition of noise in the training data, and Gaussian noise is generally used. However, a Gaussian distribution is unsuitable for training with RSSI data in which the noise intensity varies with distance. Therefore, in this study, we proposed three methods for varying the SNR according to the distance: (*i*) multiplying the proportional factor, (*ii*) adjusting the probability of noise occurrence, and (*iii*) adjusting the standard deviation of the noise distribution. Subsequently, we compared their performances with that of a model using general Gaussian noise.

## 2. Related Work

Numerous studies have been conducted to improve indoor positioning accuracy using various wireless signals. In [13], the authors designed a probabilistic framework using a data structure named conditioned trajectory graph, which showed trajectories and priority probabilities compressively. In [14], the authors developed a Bayesian inference and design sequential sampler that had high accuracy and effectiveness for cleaning RFID data. In [15], to solve the problem of matching a sequence of locations, they estimated a probability distribution function that accorded with the reading values, with two conditions: the valid movement of the object and the capacity of the positions. In [16], the authors proposed using V-track with a map-matching scheme based on an HMM and travel time estimation method which could distinguish delay-prone segments effectively and provide an accurate delay for delay-aware routing algorithms. In [17], the authors organized a probabilistic distance-aware graph with the characteristics of indoor topology, RFID readers, and designed algorithms that could identify false negatives and restore lost information in indoor RFID tracking data. In [18], the authors devised a new particle filter to enhance position estimation under an environment that utilized an RSSI from BLE beacons. This work attempted to identify a solution using a hidden Markov model. The evaluations, which were performed employing static and adaptive particle filters utilizing latent variables, showed enhancement levels of 20% to 40%.

Currently, deep-learning-based positioning systems have achieved effective performances. To consider RSSI fingerprints for use in deep neural networks, a method using a CNN by converting time-series data into a two-dimensional matrix is commonly used. The simplest method is to reshape a one-dimensional vector into a two-dimensional matrix [19]. In this study, the authors used the zero-padding method to convert the RSSI vector into a square matrix. Similarly, in [20], the input data were constructed by converting a vector into a matrix and training it with the Gaussian process regression (GPR) model, which is a probabilistic method for nonlinear regression problems. In [21], the authors used a continuous wavelet transform (CWT) to convert one-dimensional data into two-dimensional images for CNN-based fingerprint localization. In [22], a frequency-domain Gramian angular field (FDGAF) algorithm was proposed to convert the vibration signal of a flat wheel into featured images which could preserve original characteristic information.

Some studies have used RNNs for time-series fingerprints of moving targets, such as a long short-term memory (LSTM) [23]. In [24], the authors proposed a weighted average filter and compared the accuracy of RNNs such as vanilla RNN, LSTM, gated recurrent unit (GRU), bidirectional RNN (BiRNN), bidirectional LSTM (BiLSTM), and bidirectional GRU (BiGRU). However, there remained a performance aggravation problem owing to noise in the signal data. To reduce the aggravation of indoor noise, a method for removing noise from RSSI data has been proposed in several studies. The Kalman filter, a representative algorithm for removing noise from signals, improves indoor positioning performance by removing noise from RSSI data [25]. Moreover, DAE is also an algorithm used for denoising. In [26], a DAE was used to remove noise from RSSI data, and the location was estimated using KNN.

However, these methods do not consider the amount of noise variation according to the distances between the user and the anchor devices. In this study, we proposed three noise generation methods to reflect the characteristics of the RSSI data where the SNRs vary with the distance.

There are various perspectives that can solve the question of indoor positioning with using different approaches. For example, [27] investigated the time of flight (TOF) method and found a solution by using pulse-echo ultrasonic signals with TOF, and [28] focused on leveraging the distribution of RSSIs and the user’s state for a real-time localization using a deep learning approach. In [29], the authors investigated combining such methods with radar and lidar to deal with localization for a vehicle environment. In [30], the authors employed multiple infrastructure lighting spotlights and a position-sensitive device (PSD) sensor for precise indoor positioning.

Moreover, Wi-Fi has also been widely utilized to accomplish indoor positioning. For instance, [31] employed a multipath component analysis to enhance the robustness and performance for indoor positioning; however, energy consumption was a major issue, although its performance could be better compared to a BLE-based positioning solution. Until now, Wi-Fi has been compared to a BLE when implementing an indoor positioning system. In [32,33], the use of a BLE had some advantages, i.e., it was small, inexpensive, energy-efficient, and operable with battery, and thus, it could be deployed in a space such as a room with a high density and low cost, but it was not suitable for deployment in a wide area due to the coverage of its radio.

## 3. DAE-Based BLE Indoor Localization

RSSI data contain noise owing to the multipath effect and the signal interference present in an indoor environment, which degrade performance. To prevent the degradation of localization accuracy, we employed a DAE to remove noise from the RSSI data.

**Figure 2:**
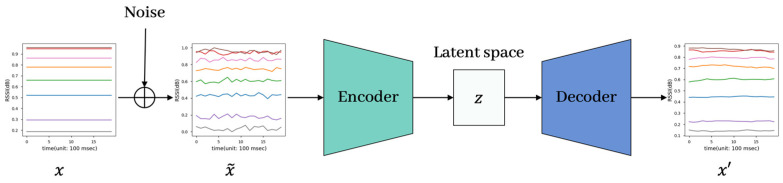
The structure and training data of the denoising autoencoder; each color line presents RSSIs from different AP.

The DAE was an unsupervised deep-learning-based denoising algorithm used for image denoising [34] and dimensionality reduction [35]. However, in escaping an image area, some studies have achieved success by applying autoencoder-based models to time series data, e.g., measurements from sensors, as used in [36,37,38]. Complying with this, our DAE was utilized to cut off noise in the proposed method. Figure 2 shows the structure of the DAE model, where x was the original training data, $\tilde{x}$ was the input data with random noise added to x, and $\tilde{x}$ was the input to the encoder that was compressed into the latent space z. Then, the decoder restored z to x. The encoder learned to extract effective features from noisy data.

**Figure 3:**
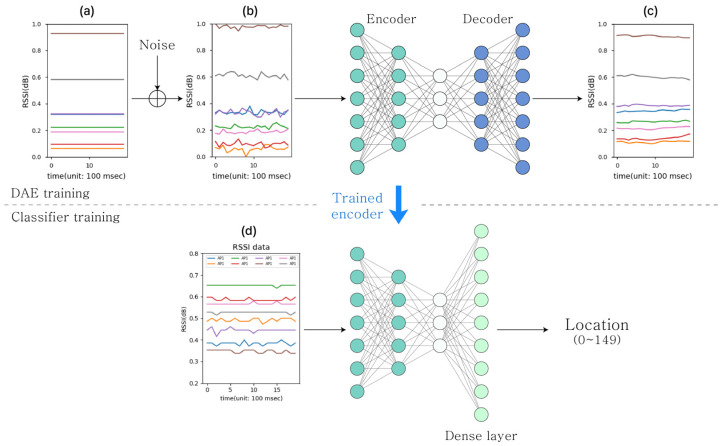
The architecture of proposed DAE-based indoor localization model.

The system architecture of the proposed indoor localization model employing the DAE is shown in Figure 3. Figure 3a–c shows the pre-training data, and Figure 3d shows the experimentally measured RSSI data. Figure 3a shows the set of constant values, i.e., pure signal data without noise, ideally, and Figure 3b shows the set of two-dimensional data created by adding the artificial random noises between [0, 1], which were depending on the window size of the RSSI data. Thus, in the DAE training step, we could determine how well the output data (shown in Figure 3c) were adequately denoised by comparing them to the pure constants (shown in Figure 3a). The pure data S can be expressed as:

(1) $$S=\left[\begin{array}{ccc} a_{11} & \cdots & a_{1\omega }\\ \vdots & \ddots & \vdots \\ a_{N1} & \cdots & a_{N\omega } \end{array}\right],$$

where N is the total number of APs and ω is the window size.

The noisy data (b) obtained by adding random noise to (a) can be expressed as:

(2) $$\tilde{S}=\left(1-\alpha \right)S+\alpha P,$$

where P is the noise and α is the noise factor. The method for generating noise is described in Section 4. The noisy data are used as the input data for the DAE, and the encoder of the DAE compresses the input data into the latent space as follows:

(3) $$z=f_{\theta }\left(\tilde{S}\right)=s\left(W\tilde{S}+b\right),$$

where $\tilde{S}\in {\left[0,1\right]}^{D}$, θ=W,b, and W is a weight matrix of the size (d×D).

The encoder and decoder consist of convolutional layers. The decoder then restores the latent space to the original data as follows:

(4) $$S^{\prime }=g_{\theta }\left(z\right)=s\left(W^{\prime }z+b^{\prime }\right),$$

where $\theta^{\prime }=W^{\prime },b^{\prime }$ and $S^{\prime }\in {\left[0,1\right]}^{D}$ is the denoised data, which corresponds to (c), which is an output of the DAE.

**Figure 4:**
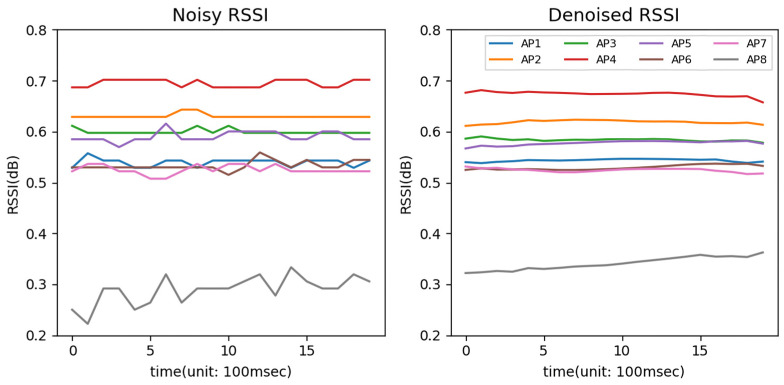
An example of noisy RSSI data and denoised RSSI data from eight APs.

The loss function of the DAE is defined as follows:

(5) $$L\left(S,S^{\prime }\right)=\frac{1}{n}\sum_{k=1}^{n}{\left\|S_{k}-{S^{\prime }}_{k}\right\|}^{2},$$

which is the mean squared error (MSE) of *S* and *S′*. Through the above steps, the DAE learned to restore noisy data to the original data. In this process, the encoder trains effective feature extraction for noisy data. After pretraining, the encoder is reused to extract the features of the RSSI data. Figure 4 shows an example of the RSSI data from eight APs and the data denoised by the decoder.

Gaussian noise is typically used for artificially noise-added pre-training data for a DAE; however, this does not sufficiently represent the noise distribution of the actual RSSI data. Therefore, in this study, we proposed three methods for generating a noise distribution to learn RSSI data effectively.

## 4. Adaptive Nosie Generation Scheme

### 4.1. Problem Statement

The RSSI of the BLE signal measured between 0 and −100 and an example of the RSSI data measured at an arbitrary location $l_{i}$ are expressed as

(6) $$r_{i}=[r_{1},\;r_{2},\;\cdots ,\;r_{N}],\;l_{i}=i,$$

where N is the number of APs.

**Figure 5:**
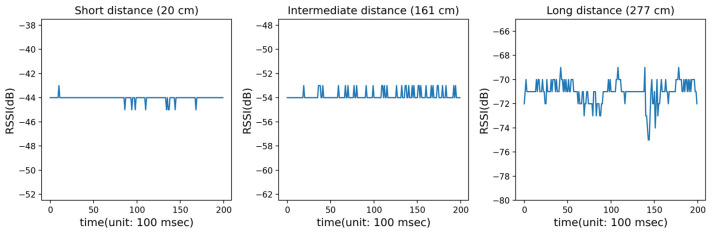
Unstable conditions of RSSI measurements according to distance increments.

The SNR of the RSSI data tended to increase as the distance between the AP and RP increased. Figure 5 shows an example of RSSI data where the distance ranges set were too short, intermediate, and far. While the RSSI values near the AP were stable, the values became irregular, and their amplitudes increased as the distance increased. From Figure 5, we can easily see that fluctuations in the RSSI values were aggravated and the peak-to-peak error range was increased.

**Figure 6:**
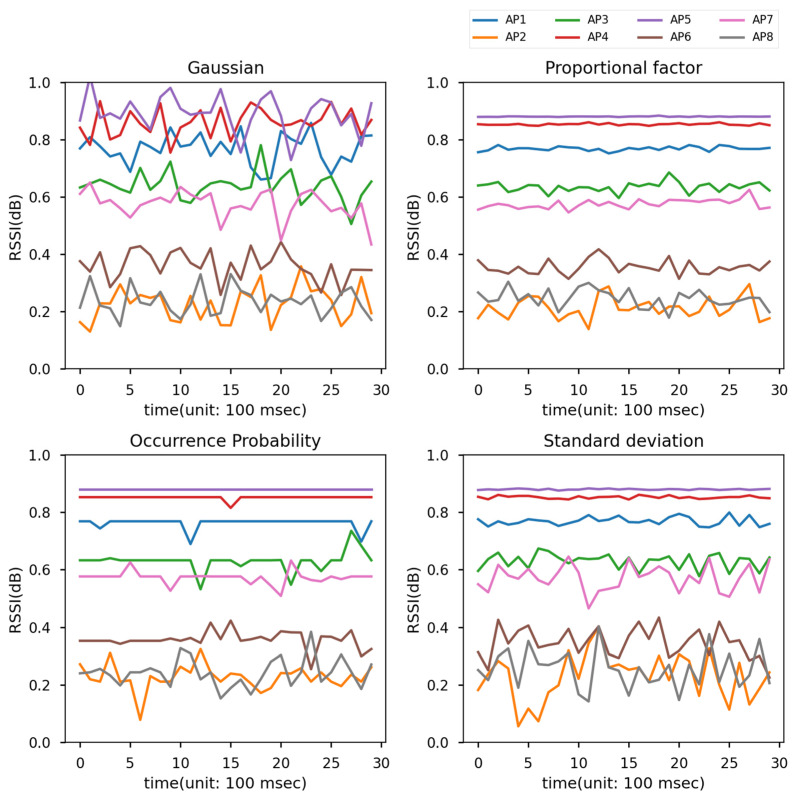
Example of test data for the DAE model generated by the Gaussian noise, proportional factor adjustment, occurrence probability adjustment, and standard deviation adjustment methods.

To adjust the SNR of the DAE training data with the distance, we proposed three methods: (*i*) adjusting the range of the noise, (*ii*) adjusting the occurrence probability of the noise, and (*iii*) adjusting the occurrence deviation of the noise. Figure 6 shows examples of noisy data generated by the proposed pre-training methods. By reflecting the proportionality of the RSSI to the pre-training data, the performance of the localization model with the DAE was improved compared to simply using Gaussian noise. We note that Section 4.2, Section 4.3 and Section 4.4 illustrate our three proposed schemes.

### 4.2. Proportional Factor Adjustment

The first method adjusted the size of the Gaussian noise according to the RSSI value by multiplying the proportional factor. This method assumed that the SNR increased with distance. The noisy data $\tilde{S}$ can be expressed as follows:

(7) $$\tilde{S}=\left(1-\alpha \right)S+\alpha P\left(x\right)\;\mathrm{and}$$

(8) $$p\left(x\right)=\frac{1}{\sigma \sqrt{2}}\exp\left(-\frac{{\left(x-\mu \right)}^{2}}{2\sigma^{2}}\right),$$

where S is the original data and α is the noise factor, and α can be denoted as follows:

(9) α=k(1−r)+ε,

where r is the RSSI data normalized to $\left[0,1\right]$, k is the proportional factor, and ε is an infinitesimal constant used to avoid the case of dividing by zero, which is configured as $0.1\times 10^{-7}$. Because the RSSI is inversely proportional to the square of the distance, $\left(1-r\right)$ is multiplied by k. The SNR of training data was adjusted by the value of k. Although this method was simple, it did not reflect the features of the discrete RSSI data.

### 4.3. Occurrence Probability Adjustment

The second method increased the probability of noise occurrence at each point in time as the distance between the AP and RP increased. The presence of noise was determined by comparing a randomly selected x with the value p at each point. The noise occurrence probability function f is expressed as follows:

(10) $$f\left(x\right)=\{\begin{array}{c} 0,\;x<p\\ 1,\;x\geq p \end{array}\;\mathrm{and}$$

(11) p=r,

where the noisy data $\tilde{S}$ is expressed as follows:

(12) $$\tilde{S}=\left(1-\alpha \right)S+\alpha P\left(x\right)f\left(x\right).$$

As p is proportional to r, the probability of adding noise increased as the distance increased. In this method, the hyper-parameter was α. This approach produced a distribution that closely resembled the actual RSSI data; however, it was difficult to tune the hyperparameter configurations.

### 4.4. Standard Deviation Adjustment

The final method adjusted the standard deviation of the Gaussian distribution. The noisy data $\tilde{S}$ can be expressed as follows:

(13) $$\tilde{S}=\left(1-\alpha \right)S+\alpha P\left(x\right)\;\mathrm{and}$$

(14) σ=1−r.

As the distance increased, the standard deviation of the Gaussian noise multiplied by the training data increased. This method could adjust the parameters of the Gaussian distribution and did not reflect the discrete properties of the RSSI values.

## 5. Evaluations

### 5.1. Experimental Environment

**Figure 7:**
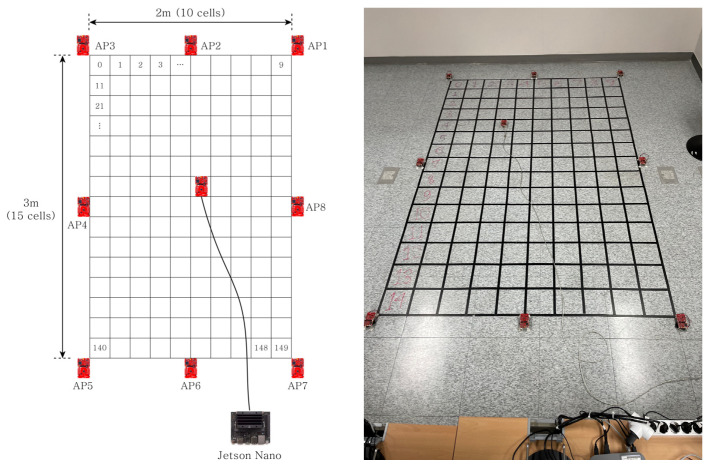
The structure of the experimental environment: (**left**) the design of the virtual environment, and (**right**) the tested experimental environment.

For the experiments, we collected RSSI data from an area of 3 m × 2 m, consisting of 150 cells divided into units of 20 cm × 20 cm. The experimental setup is shown in Figure 7. The area included 150 cells, and the localization model classified the input data into 150 classes. We measured 500 RSSI samples of BLE signals from eight APs at 100 ms intervals in every cell. We collected eight datasets over approximately three months and verified their performance on the test data. We used LAUNCHXL-CC26 × 2R1 from Texas Instruments as the beacon, sending data to an nVidia Jetson Nano via serial communication to build a dataset. The training data and the parameters of our model are described in Table 1. We note that the aim of the proposed schemes was precise indoor positioning in a small environment with stationary obstacles.

### 5.2. Analysis of Experiment Results

**Figure 8:**
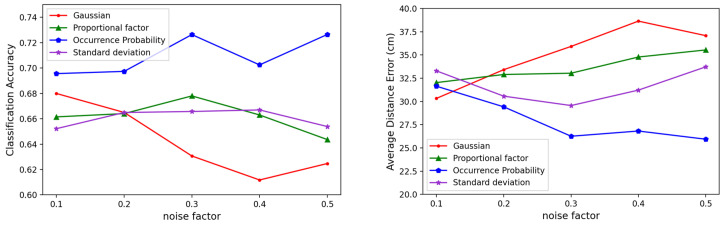
Comparison of the performances between Gaussian noise and the three noise generation methods by noise factor: (**left**) the classification accuracies, and (**right**) the average distance errors.

We compared the localization performance of our noise-generation scheme with that of Gaussian noise. To verify the results in various environments, we experimented with using different SNRs for the DAE training data. Figure 8 shows the localization accuracies and average distance errors of the models using Gaussian noise and the three proposed noise generation schemes with various noise factors. The average distance error was the Euclidean distance between the center of the classified cell and the correct cell, calculated as follows:

(15) $$Average\;Distance\;Error\left(ADR\right)=\frac{1}{K}\sum_{n=1}^{K}d_{n}\left(l,\;\hat{l}\right).$$

The model with Gaussian noise exhibited the lowest error at a noise factor of 0.1, but the error increased as the noise factor increased. In fact, the other noise schemes showed higher accuracies than the Gaussian noise at large noise intensities. This suggested that these noise generation schemes were more effective than Gaussian noise in noisy environments. In particular, the noise occurrence probability adjustment technique showed a decrease in error as the noise factor increased, and we found that it most effectively learned the noise distribution of the RSSI. 

**Figure 9:**
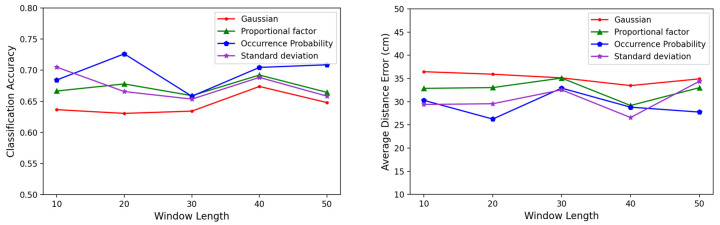
Comparison of the performances between Gaussian noise and the three noise generation methods by window length: (**right**) the classification accuracies, and (**left**) the average distance errors.

We measured the results by varying the window length of the sequence data, as shown in Figure 9. Generally, a larger window length allowed us to include more temporal information in a single dataset, resulting in improved accuracy. However, as the window size increased, more memory was used for training, and the amount of data that need to be initially input when the model was used in the real world also increased. Therefore, the window length was determined by considering the training environment and response time of the localization system. In the experiments, we found that less than 50 bytes of window length showed less than 500 ms of delay, and so we assumed these were satisfactory for the operation. Among the variable window lengths, as shown in the results in the figure, we chose 20 bytes as the optimal value, and then we applied it to the experiments that followed.

**Figure 10:**
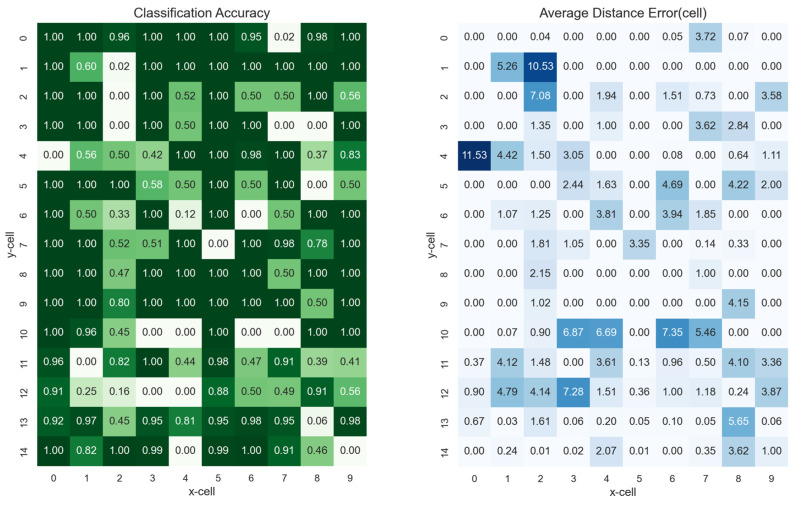
The results of each cell in experimental area: (**left**) the classification accuracies, and (**right**) the average distance errors.

Figure 10 shows the classification accuracies and errors for each cell in the experimental area. The window length and noise factor of this model were 20 and 0.5, respectively, showing the lowest error, as shown in Figure 8. The outer cells adjacent to the AP showed high accuracy whereas the other cells showed some errors. This implied that as the distance increased, the SNR due to the signal interference of the AP increased. The results showed mostly low errors, with the exception of a few cells.

**Figure 11:**
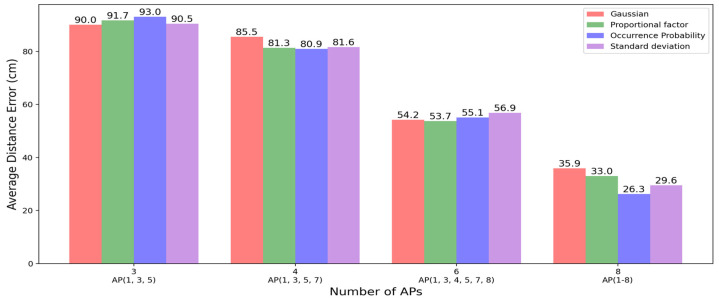
The average distance errors of the proposed models by number of APs.

To test whether our model was suitable for real-world environments, we measured its performance for cases with fewer APs, as shown in Figure 11. We compared the errors when the numbers of APs were three, four, six, and eight. The number of APs used is indicated by the label on the *x*-axis in the figure. The window length and noise factor were set to 20 and 0.5, respectively. With three or four APs, the errors were approximately 80–90 cm (four to five cells). When the number of APs increased to six, the errors decreased to 50–60 cm, and the lowest error was obtained when all eight APs were used. More APs meant more information in the data, which could lead to a better performance, but it also meant more cost to build the infrastructure in a real-world environment.

Basically, the three proposed methods were commonly aiming to create a solution for adapting RSSIs that varied according to the distance increments because the value of an RSSI can have an exponential error when the distance is increased. Referring to the previous results, the method of occurrence probability showed slightly better results overall, but it showed a poorer performance when the number of APs was small, as shown in Figure 11. Synthetically, we could make a decision that the method of occurrence probability was advantageous in an environment of densely deployed APs. Otherwise, vice versa, the methods of proportional factor and standard deviation were advantageous. Meanwhile, the three proposed methods were advantageous compared to the Gaussian method that was originally employed in the DAE model.

**Figure 12:**
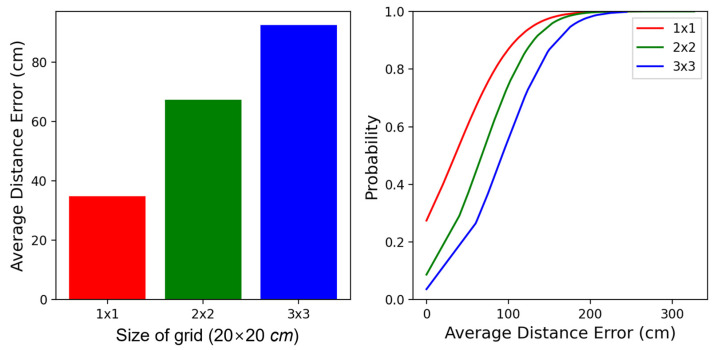
The results by the size of the cell: (**left**) the average distance errors, and (**right**) the cumulative distribution function (CDF).

Figure 12 shows the results of average distance errors and cumulative distribution function (CDF) according to the size of each cell in the same testbed environment. In this experiment, the size of a cell varied with the configurations as 1 × 1, 2 × 2, and 3 × 3, and all cells in the testbed were equivalent at each configuration of the cell size. As seen in the results shown on the left side on Figure 12, a large cell presented higher distance errors because a missed classification had a long-distance error due to the large-sized cellularization. Hence, we could not say that the policy of using lowly dense cellularization was better. Meanwhile, the results of the CDF, shown on the right side of Figure 12, presented reasonable patterns.

**Figure 13:**
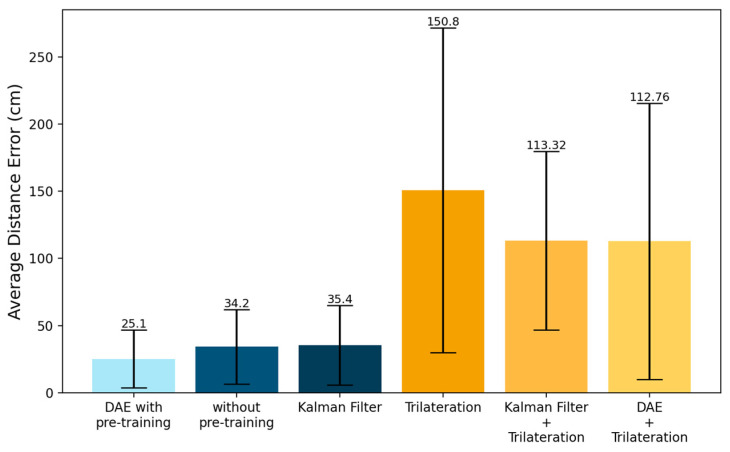
The average distance errors of the proposed models and different algorithms.

To verify the effect of denoising the RSSI data, we compared the average distance errors of several algorithms. Figure 13 shows the errors of the proposed model and different approaches, with the error range including the value of the standard error presented on the top of each error bar. Table 2 describes the results of the classification accuracies and the errors with computational efficiencies. In general, neural-network-based models require high computing resources due to the great amount of calculation such that a majority of the computational efficiency is issued. From this perspective, the results of the computational efficiencies, i.e., the FLOPS, for all of the neural-network-based approaches are shown in the right side of Table 2. We note that the presented “DAE with pre-training” in the figure and in the table represents training both the DAE and the classifier, and the presented “DAE without pre-training” represents only training the classifier with the feature obtained from the encoder layer of the DAE. The first is the proposed model and the second is the model without pre-training, and the second model consists of convolutional layers with the same structure as the encoder and dense layers of the proposed model. The RSSI data were input into the encoder layer and classified into location cells, bypassing the decoder. In this case, the encoder and decoder were not pre-trained, but the encoder was trained together with the classifier. This was an experiment to observe how pre-training the encoder and decoder affected the positioning performance. We observed that pre-training the DAE can improve the localization accuracy by denoising the RSSI.

The Kalman filter is a popular algorithm used for denoising signal data [25]. The Kalman filter was essentially designed for denoising data from a linear signal, and thus, it is not favorable to use the Kalman filter to denoise data from a non-linear signal. There is an alternative solution—the unscented Kalman filter [41]—that enhanced the original Kalman filter to be able to denoise non-linear signals. Although many people have argued about whether an RSSI meets a linear signal (or not), considering the high noise environment, we employed the unscented Kalman filter to be combined with the proposed training model. We note that the notations of the Kalman filter from here also consider the unscented Kalman filter, including the notations in Table 2.

To compare the denoising performances of the Kalman filter and the DAE, we input the RSSI data denoised by the Kalman filter into dense layers that had the same structure as the classifier of the proposed model, and then we estimated the location. The results showed that the DAE was more effective than the Kalman filter in denoising the RSSI data.

Trilateration is an algorithm that determines unknown positions using distance in localization systems, such as in GPS. This method resulted in the highest error among those algorithms because it could not remove the effect of noise from the RSSI data. We also observed the results of the denoising though the Kalman filter and the DAE, followed by estimating the locations through trilateration. As shown in Table 2, both methods had similar errors: 113.32 cm and 112.76 cm, respectively, and these errors were lower than those of trilateration without denoising. The results showed that the denoising process could enhance the localization accuracy in an indoor environment.

For further evaluation, the overall comparison information including the test environment and performance results are presented in Table 3, denoting the information of the proposed scheme in the last row. The table includes five recently published schemes that employed BLE for the indoor positioning, and these could be classified into several perspectives, i.e., algorithm or machine learning (ML), combined technology such as sensors, and so on. Focusing on the results, the proposed scheme showed a better performance with a low Tx power, without any combined technology. For a detailed description, Ref. [42] showed a poorer performance due to the large size of the cell and the large testbed, but it led to reasonable results derived from the high Tx power. References [43,44] presented high performances due to combining other physical devices such as augmented reality (AR), Wi-Fi, QR, or a MEMS sensor, and Ref. [45] employed AoA with multiple antennas. We note that the number of cells in [46] could not be presented and were denoted as N/A because they approached trilateration. In addition, from the perspective of ML, Ref. [42] used a weighted *k*-NN with SVM, Ref. [44] used a multi-layer perceptron (MLP) with a *k*-NN, and Ref. [45] used an MLP with a CNN. The highest performance without any combined technology was found in [46], but it had a relatively high standard deviation, 75 cm, compared to the proposed scheme, which presented 25.1 cm, as shown in Figure 13.

**Figure 14:**
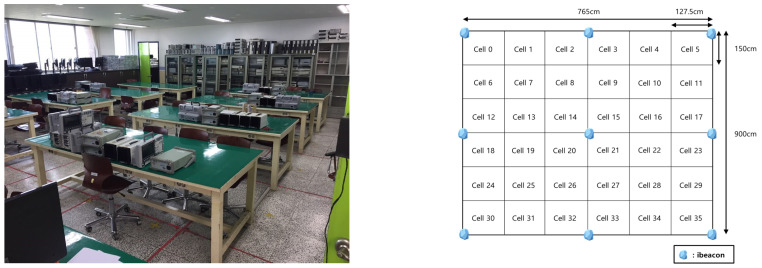
Environment of the extra experiment for the performance evaluations in a real indoor room.

For further evaluations, the proposed model was trained and tested in another experiment under an extra environment, as shown in Figure 14. In the test environment, nine APs were deployed on the roof, and each AP was configured to be 0 dBm of transmitted power. Although there were many obstacles, such as tables, chairs, cabinets, and various equipment on each table, they were stationary, in general. The overall space was classified into 36 cells ranging from cell number 0 to 35, and 324,000 numbers of RSSI data were used for training and testing, and these were measured depending on each cell, i.e., 1000 RSSIs × 9 APs × 36 cells. With the data, the training and testing processes again progressed, but the details of the parameter configurations were equivalently applied based on Table 1.

**Figure 15:**
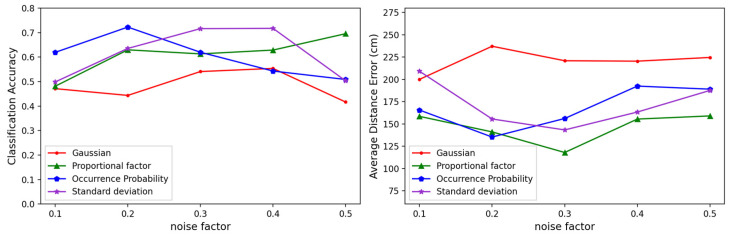
Comparison of the performances of Gaussian noise and the three noise generation methods by noise factor in the extra experiment for a real indoor room: (**left**) the classification accuracies, and (**right**) the average distance errors.

The number of classes were not equivalent compared with the previous testbed shown in Figure 7 because it was not convenient to measure the data for such a small cell space with many obstacles. In addition, the distance covered from each AP became longer. For this reason, compared to the results from the previous testbed, the results (shown in Figure 15) showed decreased classification accuracies and increased distance errors. However, the results showed a classification accuracy of approximately 72% for the 36 classes and approximately 118 cm of distance error. Regarding that the space for the test was approximately 688,500 cm^2^, it could be satisfied to meet a reasonable performance considering the general IPS system.

## 6. Conclusions

This study proposed a variable DAE employing adaptive noise generation schemes for the effective learning of denoising an RSSI for indoor localization. The distance-dependent noise distribution of the RSSI data was considered when training the DAE model. Our models outperformed Gaussian noise as the SNR increased. The model with the noise occurrence probability adjustment method had the highest accuracy (72.6%), outperforming the other localization algorithms. Our noise generation schemes can improve localization performance in noisy indoor environments owing to signal interference and environmental changes. As a Wi-Fi environment has less dense AP deployment and higher transmission power, there may be a limited point when training a DAE using Wi-Fi due to the smaller size of the data unit and lower noise. However, the proposed schemes can be capable in any research investigating RSSI analytics for a variable purpose instead of using algorithm-based filters. In future work, we will elaborate the position-decision model after the DAE process, including the regression perspective and replacing the dense layer. In addition, we have a further plan to expand our testbed and aim at intensive noise analytics to enhance practical position accuracy with respect to the relationship between neighboring cells in a test environment that can be applied to the real world.

## Figures and Tables

**Table 1:** Configuration of the hyperparameters used to train the model.

Class	Parameters	Configurations	
		DAE	Classifier
Data	RSSI measurement	50 ms/AP	-
	No. of samples	100,000	516,856
	Ratio (train:val:test)	7:2:1	8:1:1
	Window length	10/20/30/40/50	10/20/30/40/50
	No. of APs	3/4/6/8	3/4/6/8
	Input size	(100,000, ω ^1^, N ^2^, 1)	(100,000, ω×N)
Learning	Loss Function	MSE	Categorical cross entropy
	Optimizer	Adam	Adam
	Learning rate	0.001	0.001
	Batch size	32	32
	Data arrangement	Shuffled (train:val)	Shuffled (train:val)
	Activator (hidden)	ReLU	ReLU
	Activator (output)	Sigmoid	Softmax
	Normalization	None	None
	Epoch	300	500
	Training time	20 s/step	70 s/step
	No. of layers	12	2
	No. of class	-	150

^1^ window length, ^2^ number of APs.

**Table 2:** Results of the experiments comparing the different algorithms’ performances.

Algorithms	Classification Accuracy	ADE (cm)	FLOPS (G)
DAE with pre-training	0.74	25.10	7137.00
DAE without pre-training	0.64	34.20	3556.59
Kalman filter plus classifier	0.62	35.40	49.90
Trilateration [39]	-	150.80	-
Kalman filter plus trilateration [40]	-	113.32	-
DAE plus trilateration	-	112.76	7087.10

**Table 3:** Comparison results of state-of-the-art schemes employing BLEs for indoor positioning.

Target Schemes	Year	Basic Method	BLE Version	Combined Tech.	Testbed Size (m^2^)	Sample Rate	No. of Cells	No. of APs	Tx Power	Distance Error
[43]	2022	Algorithm	4.0	AR	20 × 17	10–30 Hz	N/A	12	Unknown	<10 cm
[42]	2022	ML	5.0	-	80 × 35	Unknown	200	60	4 dBm	570 cm
[44]	2023	ML	5.0	Wi-Fi, QR, or MEMS	Middle	5 Hz	Unknown	Unknown	Unknown	52 cm
[45]	2022	ML	≥4.0	AoA	7 × 14 simulation	Unknown	2450	4	Unknown	60 cm
[46]	2022	Algorithm	5.0	2 × BLE	6.5 × 7.6	20 Hz	N/A	5	Unknown	24 cm
Pre-trained DAE	2023	ML	5.2	-	2 × 3 9 × 7.65	20 Hz	150	8	−5 dBm	25.1 cm 118 cm

## Data Availability

Not applicable.

## References

[1] Liu H., Darabi H., Banerjee P., Liu J. (2007). Survey of wireless indoor positioning techniques and systems. IEEE Trans. Syst..

[2] Al-Ammar M.A., Alhadhrami S., Al-Salman A., Alarifi A., Al-Khalifa H.S., Alnafessah A., Alsaleh M. Comparative survey of indoor positioning technologies, techniques, and algorithms. Proceedings of the International Conference on Cyberworlds.

[3] Zho B.D., Kwon S.O., Cheon S.E. (2017). Indoor positioning system using ultrasonic and RF. J. Korea Inst. Inf. Sci..

[4] Gu Y., Lo A., Niemeyer’s I. (2009). A survey of indoor positioning systems for wireless personal networks. IEEE Commun. Surv. Tutor..

[5] Zhou J., Zhang H., Mo L. Two-dimension localization of passive RFID tags using AOA estimation. Proceedings of the 2011 IEEE International Instrumentation and Measurement Technology Conference.

[6] Batstone K., Oskarsson M., Åström K. Robust time-of-arrival self calibration and indoor localization using Wi-Fi round-trip time measurements. Proceedings of the IEEE International Conference on Communications Workshops (ICC).

[7] Jeong W., Lim J., Baek H., Koo J. (2022). TDOA/AOA-Based Unknown Surveillance Radar Localization Scheme Using a Single UAV. J. KICS.

[8] Kim J., Lim J., Baek H. (2022). RSS/AOA Positioning Scheme Using Multiple Receivers in Indoor VLC Communications. J. KICS.

[9] Yiu S., Dashti M., Claussen H., Perez-Cruz F. (2017). Wireless RSSI fingerprinting localization. Signal Process..

[10] Xiao Z., Wen H., Markham A., Trigoni N. Lightweight map matching for indoor localisation using conditional random fields. Proceedings of the 13th International Symposium on Information Processing in Sensor Networks.

[11] Fazzinga B., Flesca S., Furfaro F., Parisi F. Offline cleaning of RFID trajectory data. Proceedings of the 26th International Conference on Scientific and Statistical Database Management.

[12] Vincent P., Larochelle H., Bengio Y., Manzagol P.A. Extracting and composing robust features with denoising autoencoders. Proceedings of the 25th International Conference on Machine Learning.

[13] Fazzinga B., Flesca S., Furfaro F., Parisi F. (2016). Exploiting integrity constraints for cleaning trajectories of RFID-monitored objects. ACM Trans. Database Syst. (TODS).

[14] Zhao Z., Ng W. A model-based approach for RFID data stream cleansing. Proceedings of the 21st ACM International Conference on Information and knowledge management.

[15] Fazzinga B., Flesca S., Furfaro F., Parisi F. (2020). Interpreting RFID tracking data for simultaneously moving objects: An offline sampling-based approach. Expert Syst. Appl..

[16] Thiagarajan A., Ravindranath L., LaCurts K., Madden S., Balakrishnan H., Toledo S., Eriksson J. VTrack: Accurate, energy-aware road traffic delay estimation using mobile phones. Proceedings of the 7th ACM Conference on Embedded Networked Sensor Systems.

[17] Baba A.I., Lu H., Pedersen T.B., Xie X. Handling False Negatives in Indoor RFID Data. Proceedings of the IEEE 15th International Conference on Mobile Data Management.

[18] Daniş F.S. Live RSSI Filtering for Indoor Positioning with Bluetooth Low-Energy. Proceedings of the IEEE 12th International Conference on Indoor Positioning and Indoor Navigation (IPIN).

[19] Mazlan A.B., Ng Y.H., Tan C.K. (2022). A Fast Indoor Positioning Using a Knowledge-Distilled Convolutional Neural Network (KD-CNN). IEEE Access.

[20] Zhang G., Wang P., Chen H., Zhang L. (2019). Wireless indoor localization using convolutional neural network and Gaussian process regression. Sensors.

[21] Soro B., Lee C. (2019). Joint Time-Frequency RSSI Features for Convolutional Neural Network-Based Indoor Fingerprinting Localization. IEEE Access.

[22] Bai Y., Yang J., Wang J., Li Q. (2020). Intelligent diagnosis for railway wheel flat using frequency-domain Gramian angular field and transfer learning network. IEEE Access.

[23] Xu B., Zhu X., Zhu H. (2019). An efficient indoor localization method based on the long short-term memory recurrent neuron network. IEEE Access.

[24] Hoang M.T., Yuen B., Dong X., Lu T., Westendorp R., Reddy K. (2019). Recurrent neural networks for accurate RSSI indoor localization. IEEE Internet Things J..

[25] Ali-Loytty S., Perala T., Honkavirta V., Piché R. Fingerprint Kalman filter in indoor positioning applications. Proceedings of the IEEE Control Applications, (CCA) & Intelligent Control, (ISIC).

[26] Xiao C., Yang D., Chen Z., Tan G. (2017). 3-D BLE indoor localization based on denoising autoencoder. IEEE Access.

[27] Pullano S.A., Bianco M.G., Critello D.C., Menniti M., La Gatta A., Fiorillo A.S. (2020). A Recursive algorithm for indoor positioning using pulse-echo ultrasonic signals. Sensors.

[28] Nabati M., Ghorashi S.A. (2023). A real-time fingerprint-based indoor positioning using deep learning and preceding states. Expert Syst. Appl..

[29] Burnett K., Wu Y., Yoon D.J., Schoellig A.P., Barfoot T.D. (2023). Are we ready for radar to replace lidar in all-weather mapping and localization?. IEEE Robot. Autom. Lett..

[30] De-La-Llana-Calvo Á., Lázaro-Galilea J.L., Alcázar-Fernández A., Gardel-Vicente A., Bravo-Muñoz I., Iamnitchi A. (2022). Accuracy and Precision of Agents Orientation in an Indoor Positioning System Using Multiple Infrastructure Lighting Spotlights and a PSD Sensor. Sensors.

[31] Zayets A., Steinbach E. Robust WiFi-based indoor localization using multipath component analysis. Proceedings of the International Conference on Indoor Positioning and Indoor Navigation (IPIN).

[32] Luo R.C., Hsiao T.J. (2019). Indoor localization system based on hybrid Wi-Fi/BLE and hierarchical topological fingerprinting approach. IEEE Trans. Veh. Technol..

[33] Montoliu R., Sansano E., Gascó A., Belmonte O., Caballer A. (2020). Indoor positioning for monitoring older adults at home: Wi-Fi and BLE technologies in real scenarios. Electronics.

[34] Gondara L. Medical image denoising using convolutional denoising autoencoders. Proceedings of the IEEE 16th International Conference on Data Mining Workshops (ICDMW).

[35] Dasan E., Panneerselvam I. (2021). A novel dimensionality reduction approach for ECG signal via convolutional denoising autoencoder with LSTM. Biomed. Signal Process. Control.

[36] Zhang H., Liu K., Shang Q., Feng L., Chen C., Wu Z., Guo S. Dual-band wi-fi based indoor localization via stacked denosing autoencoder. Proceedings of the IEEE Global Communications Conference (GLOBECOM).

[37] Yin C., Zhang S., Wang J., Xiong N.N. (2020). Anomaly detection based on convolutional recurrent autoencoder for IoT time series. IEEE Trans. Syst. Man Cybern. Syst..

[38] Thill M., Konen W., Wang H., Bäck T. (2021). Temporal convolutional autoencoder for unsupervised anomaly detection in time series. Appl. Soft Comput..

[39] Yang B., Guo L., Guo R., Zhao M., Zhao T. (2020). A novel trilateration algorithm for RSSI-based indoor localization. IEEE Sens. J..

[40] Cantón Paterna V., Calveras Auge A., Paradells Aspas J., Perez Bullones M.A. (2017). A bluetooth low energy indoor positioning system with channel diversity, weighted trilateration and kalman filtering. Sensors.

[41] Wan E.A., Van Der Merwe R. The unscented Kalman filter for nonlinear estimation. Proceedings of the IEEE Adaptive Systems for Signal Processing, Communications, and Control Symposium.

[42] Aranda F.J., Parralejo F., Álvarez F.J., Paredes J.A. (2022). Performance analysis of fingerprinting indoor positioning methods with BLE. Expert Syst. Appl..

[43] Daniş F.S., Naskali A.T., Cemgil A.T., Ersoy C. (2022). An indoor localization dataset and data collection framework with high precision position annotation. Pervasive Mob. Comput..

[44] Yu Y., Zhang Y., Chen L., Chen R. (2023). Intelligent Fusion Structure for Wi-Fi/BLE/QR/MEMS Sensor-Based Indoor Localization. Remote Sens..

[45] Koutris A., Siozos T., Kopsinis Y., Pikrakis A., Merk T., Mahlig M., Papaharalabos S., Karlsson P. (2022). Deep Learning-Based Indoor Localization Using Multi-View BLE Signal. Sensors.

[46] Drozd S., Tomlain J., Marko M., Teren O., Tomlain J. Evaluation of the Cost-Effective Indoor Wireless Positioning System Using RSSI Method. Proceedings of the New Trends in Signal Processing (NTSP).

